# Characterization of a new podovirus infecting *Paenibacillus larvae*

**DOI:** 10.1038/s41598-019-56699-y

**Published:** 2019-12-30

**Authors:** Henrique G. Ribeiro, Luís D. R. Melo, Hugo Oliveira, Maarten Boon, Rob Lavigne, Jean-Paul Noben, Joana Azeredo, Ana Oliveira

**Affiliations:** 1 CEB - Centre of Biological Engineering, LIBRO - Laboratório de Investigação em Biofilmes Rosário Oliveira, University of Minho, 4710-057 Braga, Portugal; 2 Laboratory of Gene Technology, KU Leuven, Leuven, Belgium; 3 Biomedical Research Institute and Transnational University Limburg, Hasselt University, Agoralaan D, 3590 Hasselt, Belgium

**Keywords:** Environmental biotechnology, Pharmacology

## Abstract

The *Paenibacillus larvae* infecting phage API480 (vB_PlaP_API480) is the first reported podovirus for this bacterial species, with an 58 nm icosahedral capsid and a 12 × 8 nm short, non-contractile tail. API480 encodes 77 coding sequences (CDSs) on its 45,026 bp dsDNA genome, of which 47 were confirmed using mass spectrometry. This phage has got very limited genomic and proteomic similarity to any other known ones registered in public databases, including *P. larvae* phages. Comparative genomics indicates API480 is a new species as it’s a singleton with 28 unique proteins. Interestingly, the lysis module is highly conserved among *P. larvae* phages, containing a predicted endolysin and two putative holins. The well kept overall genomic organisation (from the structural and morphogenetic modules to the host lysis, DNA replication and metabolism related proteins) confirms a common evolutionary ancestor among *P. larvae* infecting phages. API480 is able to infect 69% of the 61 field strains with an ERIC I genotype, as well as ERIC II strains. Furthermore, this phage is very stable when exposed to high glucose concentrations and to larval gastrointestinal conditions. This highly-specific phage, with its broad lytic activity and stability in hive conditions, might potentially be used in the biocontrol of American Foulbrood (AFB).

## Introduction

American foulbrood disease (AFB) is one of the most devastating bacterial diseases affecting honeybees and it is caused by *Paenibacillus larvae*, a Gram-positive worldwide-distributed spore forming bacterium. This infection begins when adult bees provide spore-contaminated food to larvae in the initial stages of development (first 36 hours after egg hatching) causing larvae death^1,2^.

The burning of hives and contaminated material is the compulsory action recommended by authorities to control the proliferation of AFB which causes devastating economic loss to the beekeeping industry and the environment. The use of antibiotics is not advised as they are not active against spores, and they cause further wide-spreading bacterial resistances^3,4^. They are also forbidden in Europe (Regulation (EEC) 2377/90 and further amendments).

Bacteriophages (phages) are now valuable solutions to this infection control. Phages are bacterial viruses that specifically infect their hosts, relying on the cell biosynthetic machinery to produce new viral particles. Phages are considered self-dosing and self-limiting antibacterial agents. After the host lysis, new phages are able to trigger new infection cycles to the surrounding hosts, resulting in exponential phage growth until no host is available^5^. An important feature of phages is their inactivity in the extracellular environment, thus being innocuous to animals or plants^6^. The potential of *P. larvae* phages as a tool for treating AFB has been explored by evaluating its efficacy both in infected laboratory-raised larvae^7–9^ and in infected experimental hives^10^. Up to date, 48 *P. larvae* phage genome sequences have been described. They all belong to the *Siphoviridae* family and they mostly encode known integration genes. Their genomes have been grouped into four clusters (with Fern, Harrison, Vegas and Halcyone as representative phages) and one singleton (phage Lily), based on genomic diversity^11^. All of these 48 phages seem to have a common evolutionary ancestor, showing an overall common structure.

The isolation and genomic characterization of the first podovirus infecting *P. larvae* is reported here, together with the evaluation of its viability in experimental conditions envisaging the possibility of using this phage in AFB control.

## Results

### Phage isolation and host range

The isolation of new *P. larvae* strains was carried out in order to broaden the geographic and genetic diversity of the collection. A field sample collection carried out throughout 2018 allowed the isolation of 45 strains: 29 from hives with visible signs of infection and 16 from apparently healthy brood. All isolated strains exhibited an identical fingerprint pattern after rep-PCR matching those produced by ERIC I reference strains (data not shown).

The phage vB_PlaP_API480 (API480) was isolated from a hive soil sample collected in Guadalajara (Spain).

A panel of 68 *P. larvae* strains (including reference strains) were used to evaluate the lytic activity of API480 (Table 1). API480 revealed a broad lytic spectrum, infecting 69% of the 61 field strains, of which 57% exhibited EOP scores greater than 10%. All remaining strains (31%) were lysed from without. API480 was also able to infect the ERIC II strain CCUG 48972 (EOP < 10%) and lysed without replication one of ERIC II, one of ERIC III and two of ERIC IV strains. Only the *P. larvae* strain LMG 16252 (ERIC III) was not lysed by this phage. Additionally, lysis tests in non-*P. larvae* strains revealed that API480 was able to infect *B. circulans* and *B. coagulans*, although those strains do not propagate the phage. All the others were not sensitive to the phage, including the 1^st^ instar larvae commensal strains, *L. kunkeei* and *P. apium* alpha 2.2.

**Table 1:** API480 lytic spectra and EOP against different strains (*P. larvae* strains were obtained from honey (01), dead larvae (02) and wax (03). The EOP was scored as 0 (negative), 1 (<10%), 2 (10–100%), 3 (>100%) and LFW (lysis from without). N/A (Non-applicable).

Specie	Strain	Genotype	Score
*Paenibacillus larvae*	Pl01–03; Pl02-(23, 30b, 31, 33, 37, 46, 49, 56, 64, 66, 71, 72, 73, 74, 75, 76, 81, 84)	ERIC I	LFW
*Paenibacillus larvae*	CCUG 48973	ERIC II	LFW
*Paenibacillus larvae*	LMG 15974	ERIC III	LFW
*Paenibacillus larvae*	LMG 16247, LMG 16250	ERIC IV	LFW
*Paenibacillus larvae*	LMG 16252	ERIC III	0
*Paenibacillus larvae*	Pl02-(35, 52, 69, 77, 79, 80, 85)	ERIC I	1
*Paenibacillus larvae*	CCUG 48972	ERIC II	1
*Paenibacillus larvae*	Pl02-(07, 13, 18, 27, 51, 89)	ERIC I	2
*Paenibacillus larvae*	LMG 9820	ERIC I	2
*Paenibacillus larvae*	Pl02-(01, 07b2, 14, 21, 34, 36, 45, 47, 48, 50, 53, 54, 55, 57, 58, 59, 60, 61, 62, 63, 65, 67, 68, 70, 78, 83, 86, 87)	ERIC I	3
*Paenibacillus larvae*	Pl03–28	ERIC I	3
*Lactobacillus pentosus*	DSM 20314	N/A	0
*Lactobacillus rhamnosus*	CECT 288	N/A	0
*Lactobacillus paracasei*	CECT 277	N/A	0
*Lactobacillus casei*	CECT 5275	N/A	0
*Lactobacillus acidophilus*	ATCC 4356	N/A	0
*Lactobacillus kunkeei*	LMG 18925	N/A	0
*Bacillus subtilis*	DSMZ 10	N/A	0
*Bacillus cereus*	CEB collection	N/A	0
*Bacillus circulans*	CEB collection	N/A	LFW
*Bacillus coagulans*	CECT 12	N/A	LFW
*Parasaccharibacter apium*	Alpha 2.2	N/A	0
*Paenibacillus polymyxa*	LMG 13294	N/A	0
*Paenibacillus alvei*	LMG 13253	N/A	0

### Phage morphology

**Figure 1:**
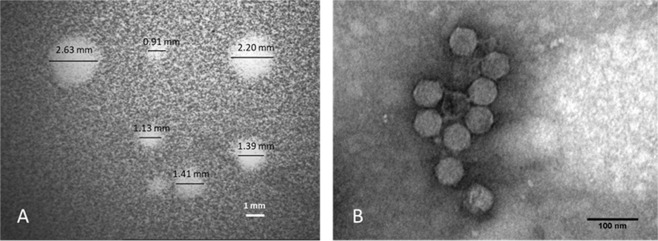
Characteristics of API480. (**A**) Plaque morphology (black lines indicate the diameter of API480 plaques obtained through a SZ40 Zoom Stereo Microscope (Olympus). Scale bar: 1 mm; (**B**) Transmission electron micrographs showing the virion particle morphology (stained with 2% uranyl acetate). Scale bar: 100 nm.

API480 forms clear plaques with diameters ranging from 0.9 to 2.6 mm, in 0.4% (w/v) agar plates (Fig. 1A). TEM images revealed the presence of phage particles with 58 nm diameter icosahedral capsids and 12 × 8 nm short non-contractile tails, belonging to the *Podoviridae* family (Fig. 1B).

### Phage genomic and proteomic properties

#### General overview

**Figure 2:**
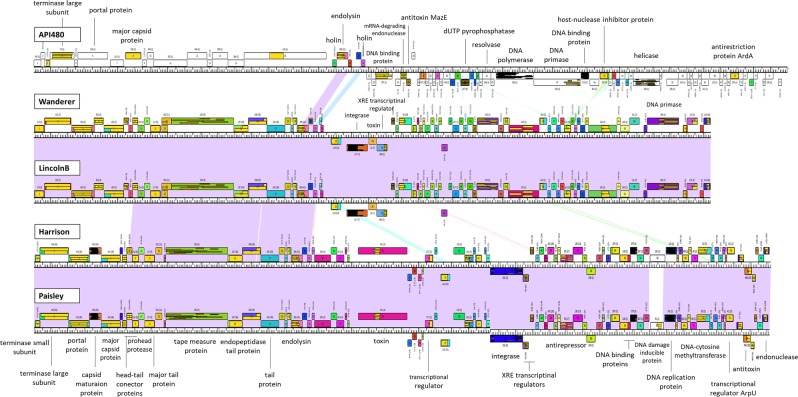
Pairwise genome maps. API480 whole-genome was compared with the closest relatives, Wanderer, LincolnB, Harrison and Paisley. Maps were created with Phamerator. Pairwise sequence similarity (minimal BLASTN cut-off E-value is 10^−5^) is indicated according to colour spectrum where purple and red lines denote regions of highest and lowest nucleotide similarity, respectively. Gene products are labelled with predicted function (phams i.e. proteins members have the same colour, orphams i.e. unique proteins are shown in white). Their positioning either above or below the bar correspond to rightwards or leftwards transcription, respectively.

Phage API480 genome, deposited in the GenBank with the accession number MK533143, is a linear dsDNA molecule of 45,026 bp with 39.24% GC content. API480 encodes 77 coding sequences (CDSs), of which 60 have hypothetical function (being 28 unique to this phage) and only 17 with an assigned function (Supplementary Table S2). Genes are tightly packed achieving 1.71 genes per 1,000 bp, with the genome being 91.9% coded. Furthermore, API480’s genome has a translation of 65 proteins that start on ATG codon (84.4%), six on GTG codon (7.8%) and six on TTG codon (7.8%). Although no tRNA or antibiotic resistance genes were identified, ten promoters and eight factor-independent terminators were found, as well as components of the MazEF toxin-antitoxin module, mRNA-degrading endonuclease (gp26) toxin MazF and its antitoxin the MazE (gp27). The API480 genome is composed by a left-to-right followed by a right-to-left transcription module (Fig. 2). The DNA packaging and phage morphogenesis genes are located at the beginning of the left arm, similar to the *P. larvae* siphoviruses. Only three proteins with assigned function were identified in this region: terminase large subunit (gp4), portal protein (gp6) and the major capsid protein (gp8). The host lysis proteins are located in the middle of the genome. The endolysin (gp18) is predicted to function as a N-acetylmuramoyl-L-alanine amidase. There are two predicted holins downstream and upstream the endolysin, both with transmembrane domains. Homologs of these putative holin_bhlA (gp17) and the phage_holin_5 (gp21) have been found in most of the *P. larvae* siphoviruses proteins^11^. The DNA replication, transcription, and metabolism are located in the right arm of the API480 genome: predicted DNA binding protein (gp24), mRNA-degrading endonuclease (gp26), antitoxin MazE (gp27), dUTP pyrophosphatase (gp39), resolvase (gp44), DNA polymerase (gp46), DNA primase (gp47), single-stranded DNA-binding protein (gp48), host nuclease inhibitor protein (gp50), helicase (gp57) and antirestriction protein ArdA (gp70). A similar organization is also observed in the other *P. larvae* phages, for instance, in Wanderer, LincolnB, Harrison and Paisley (Fig. 2), with the exception of the lysogeny module, which was not identified in the API480 genome.

#### Comparative analysis

An initial API480 genome alignment by BLASTN revealed that API480 genome is distinct from other available genomes in the GenBank. The highest genome coverage obtained to an E-value of 0.0 was only 5% by LincolnB and Wanderer with an identity of 85.6% and 89.3%, respectively. As mentioned previously, the levels of amino acid identity of API480-encoding proteins are very limited and when existent they are mostly found against *P. larvae* phage proteins (<55% average amino acid identity), indicating the lack of a close relationship between API480 and any phage currently known.

**Figure 3:**
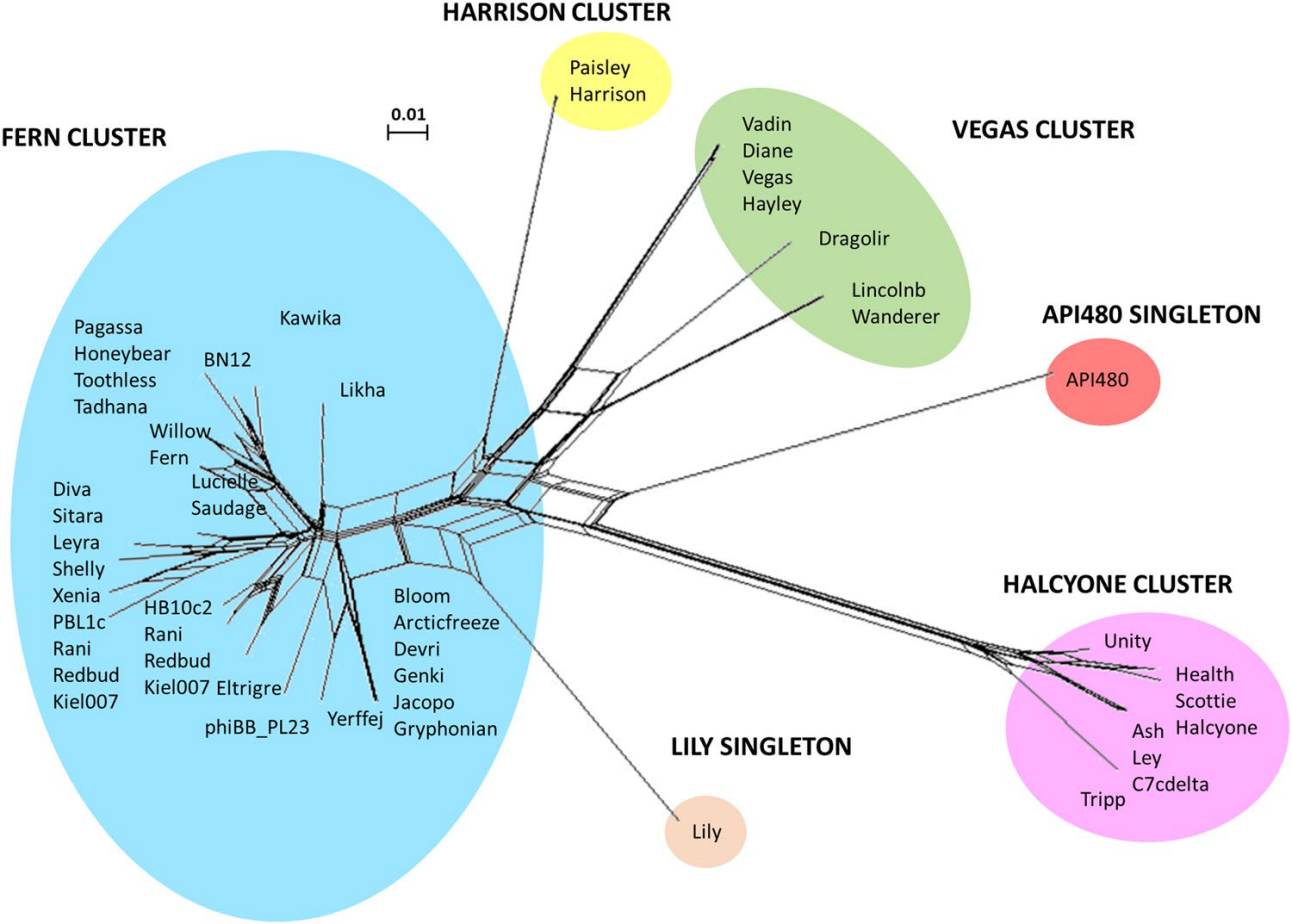
Diversity of *P. larvae* phages genomes. A total of 49 *P. larvae* phages (48 siphoviruses and 1 podovirus – API480) were compared with Phamerator in 3D and the relationship of shared gene content was visualized into 2D space with Splitstree. Clusters assigned based on sharing >40% gene products are highlighted with colours.

To improve the placement of the API480 genome in the context of the *P. larvae* phage population, we compared all *P. larvae* phages through shared gene content. Comparative analysis of all 49 *P. larvae* phage genomes resulted in the establishment of a new singleton enclosing API480 among four clusters (Fern, Harrison, Vegas and Halcyone) and one singleton (Lily), previously described by Stamereilers *et al*.^11^. The four clusters and the two singletons are shown on Fig. 3 and their relationship was represented by the branch lengths. This clustering joins phages that share more than 40% of their proteins with all members of the same cluster (Supplementary Table S3). While Lily still shares a maximum of 39% of its proteins with some phages (Arcticfreeze, Devri, Bloom, Jacopo, Genki and Gryphonian), API480 is an even more distant phage as its only shares 14% or fewer proteins. Wanderer (13.6%), LincolnB (13.6%), Harrison (11.2%) and Paisley (11.2%) can be considered as the closest relatives (Supplementary Table S3).

Through the Phamerator analysis it was also possible to see that only 19 out of the 77 predicted API480 proteins are shared with *P. larvae* siphoviruses (Supplementary Table S4) and that Harrison and Vegas are the most closely related clusters to the API480. While no universal proteins were found, it is clear that the host lysis proteins (holin_bhlA, endolysin and the holin) are mostly conserved. This is evidenced by the presence of these proteins in all phages from different clusters and singletons, except for Halcyone cluster (Supplementary Table S4). It is also visible in the Phamerator genome map that the only area highlighted in purple shading between genomes (nucleotide sequence most similar), refers to the endolysin site (Fig. 2).

#### Mass spectrometry

To confirm API480 gene predictions, its proteome was analysed by mass spectrometry. An SDS-PAGE gel loaded with unpurified phage particles showed several bands (Supplementary Fig. S1). Thirteen gel fragments were extracted and further sequenced. A total of 47 proteins could be identified (Table 2). From the 28 unique proteins from API480, 11 revealed an amino acidic sequence coverage ranging from 24.3% to 69.5%. A total of 29 proteins presented homology to bacterial proteins, from which 20 had a cover range between 7.9% and 93.7%. Finally, from the 20 proteins with homology to other phage, 16 displayed a cover range between 12.5% and 89.2%.

**Table 2:** Bacteriophage API480 proteins identified by ESI-MS/MS, after denaturation and phage particle fractionation on SDS-PAGE gel. Identified phage proteins are listed below. SDS-PAGE gel band in which the proteins were identified have been indicated as well as the protein mass, the number of identified unique peptides and the protein sequence that is covered by the peptide (in %).

Protein	Identified Function	Band no. (most abundant)	Protein MW (kDa)	No. of unique peptides	Sequence coverage (%)
gp1		11,12 (11)	16.55	6	50.40
gp2		11,12 (12)	9.96	4	58.30
gp3		5,12 (12)	11.95	6	55.90
gp4	Terminase large subunit	1,2,3,4,5,6,8,9,10,11 (1)	55.47	14	36.66
gp5		1,2,5,10,11,12 (12)	7.05	3	38.10
gp6	Portal protein	1,2,3,4,5,6,7,8,9,10,11,12 (3)	68.14	35	62.68
gp7		1,2,3,4,5,6,7,8,9,10,11,12 (6)	31.45	31	93.70
gp8	Major capsid protein	1,2,3,4,5,6,7,8,9,10,11,12 (7)	34.38	34	89.16
gp9		5,9,12,13 (12)	9.88	7	67.80
gp10		11,12,13 (12)	14.40	5	67.50
gp11		1,2,3,4,12(3)	75.23	12	24.26
gp12		1,2,3,4,5,6,8,9,11 (5)	43.56	17	50.75
gp13		12	11.64	7	68.00
gp14		2,6,7,8,9,10,11 (6)	35.47	18	55.71
gp15		6,7,8,9,10,11,12 (6)	37.05	19	63.00
gp16		1,2,3,4,5,6,7,8,9,10,11,12 (2)	128.32	71	60.75
gp17	Holin	12	9.95	4	35.00
gp20		12	12.52	3	42.90
gp21	Holin	12	8.27	1	26.60
gp24	DNA binding protein	1,2,6,9,12 (1)	36.60	5	15.58
gp26	mRNA-degrading endonuclease	12	12.52	3	28.40
gp30		11	14.13	1	13.70
gp34		12	9.76	1	16.30
gp36		10	14.43	1	7.94
gp37		12	10.62	3	59.40
gp38		12	12.33	2	37.40
gp39	Deoxyuridine 5′-triphosphate nucleotidohydrolase	1,2,3,4,5,6,8,9,10,12 (10)	18.21	7	49.69
gp40		10,11,12 (12)	9.82	6	66.30
gp43		12	7.15	5	87.90
gp44	Resolvase	1,2,3,4,5,6,7,8,9,10,11,12 (11)	19.12	8	56.90
gp46	DNA polymerase I	1,2,3,4 (3)	87.14	28	42.96
gp47	DNA primase	1,2,3 (1)	105.39	9	12.50
gp48	Dingle-stranded DNA-binding protein	3,5,8,9,10,11,12 (10)	17.60	11	62.10
gp49		1,8,9,10,11,12 (9)	20.18	13	69.48
gp50	Host-nuclease inhibitor	8,9,10 (8)	21,75	10	60.99
gp53		11,12 (12)	8.58	1	18.40
gp54		12	11.83	3	41.20
gp55		12	6.91	2	53.30
gp57	DEAD/DEAH box helicase	1,2,4,8 (1)	60.63	7	20.10
gp59		8	25,95	7	44.70
gp60		11,12 (11)	19.76	3	18.00
gp63		12	9.65	2	31.30
gp64		12	7.95	2	37.30
gp65		1,11,12 (11)	15.46	6	65.39
gp70	Antirestriction protein ArdA	9	18,92	3	19.90
gp76		1,11,12 (11)	17.36	7	59.60
gp77		12,13 (12)	8.14	4	63.40

### Phage potential for biocontrol application in hives

The potential of the therapeutic use of API480 in apiaries, as far as AFB control is concerned, was investigated through the assessment of the phage growth dynamic, life cycle and stability on field conditions.

#### Phage integration assays

Although no integrase was identified in API480 genome by the *in silico* analysis, the ability of the phage to lysogenise its host was investigated to obtain more consistent information about the phage cycle. Assuming that a lysogenized host might become resistant to the recently integrated phage by acquiring phage genetic material, the infectivity of API480 was assessed after lysogeny induction together with the presence of phage genes in host resistant colonies.

Contrary to the original Pl02–27 strain, results revealed that the phage lost the ability to infect R-Pl27 strains, and that the DNA of the same strains allowed the amplification of a 227 bp band (specific for API480) by PCR (Supplementary Fig. S3).

To assess if the use of a phage cocktail could be relevant in supporting API480 in AFB infections, the activity of the other five *P. larvae* phages from our collection against R-Pl27 colonies was investigated and all of them were infective.

#### Phage infection parameters

**Figure 4:**
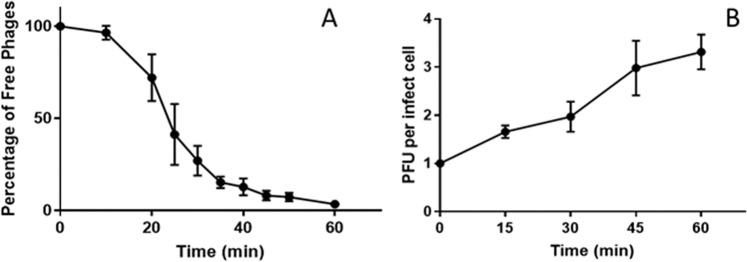
Phage-host interaction parameters. (**A**) Percentage of free API480 phages after infection of *P. larvae* (MOI = 0.1). (**B**) One-step growth curve of phage API480 in *P. larvae* Pl02–27. Shown are the PFU per infected cell. Each point represents the average of three independent assays and error bars indicate the standard deviation. Statistical significance, p < 0.05.

The assessment of the phage generation time and the phage population growth level was accomplished through the determination of phage infection parameters. Adsorption assays revealed that during the first minutes of phage contact with its host, the number of free phages rapidly decreased. After 35 min approximately 85% of the total API480 phage particles were adsorbed to its host (Fig. 4A).

As far as phage growth cycle parameters are concerned, the calculated latent period was approximately 30 min and the phage burst size was approximately 3 PFU per infected cell (Fig. 4B).

#### Phage stability in simulated field conditions

The API480 phage stability in a 50% (w/v) sucrose solution was assessed *in vitro* for 24 hours. The results revealed that, at least in this time period, the phage viability was not impacted (Supplementary Fig. S3).

**Figure 5:**
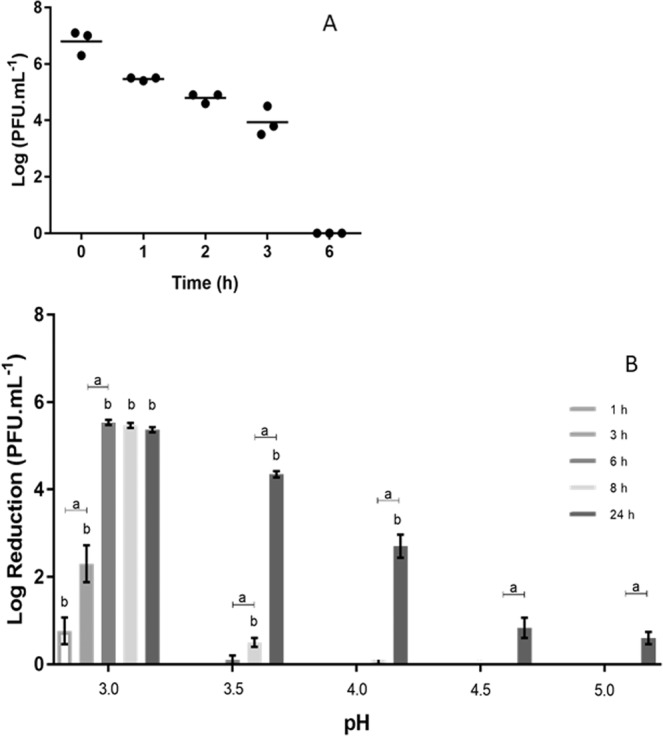
(**A**) Effect of commercial RJ on the stability of API480 (PFU.mL^−1^). Data show each of three independent assays. Limit of detection (LOD) = 3 Log; Statistical significance, p < 0.05. (**B**) Effect of pH (from 3.0 to 5.0) in API480 phage concentration (PFU.mL^−1^). Each column represents the average of three independent assays and error bars indicate the standard deviation. LOD = 1 Log; Statistical significance, p < 0.05; “a” indicates differences for the same pH; for each timepoint, “b” indicates differences between data from a given pH and the subsequent pH value.

The evaluation of phage viability in RJ (pH 4.0) revealed a total loss of phage infectivity after 6 hours (Fig. 5A).

The impact of low pH solutions (3.0, 3.5, 4.0, 4.5 and 5.0) in API480 stability was also assessed. Results, presented in Fig. 5B, revealed that after 6 hours the phage was completely inactivated at pH 3.0 (5.5 Log reduction in average). Interestingly, at pH 3.5, at the same time point, it only caused a slight decrease of 0.1 Log PFU.mL^−1^, being accentuated (p < 0.05) after 24 hours, with an average reduction of 4.3 Log PFU.mL^−1^. No effect was observed with the other pH values when assessed for periods lower than 24 hours. Nevertheless, at this time point, the average phage lost was of 2.7, 0.8 and 0.6 Log PFU.mL^−1^ at pH 4.0, 4.5 and 5.0, respectively.

The assessment of the phage stability in larvae homogenised revealed that a slight and not statistically meaningful phage reduction of 0.4 Log PFU.mL^−1^ (p > 0.05) was observed, and only after 24 h.

## Discussion

The isolation of the first genomic sequence of a *P. larvae* podovirus, vB_PlaP_API480 (API480) is described here. So far, all *P. larvae* phages (n = 48) have been reported to be siphoviruses, with a temperate lifestyle and the majority (n = 40) with a wide genetic similarity. The only exception is the Halcyone cluster (n = 8) that is quite diverse from other *P. larvae* phages^11^. We showed that API480 has very limited genomic and proteomic relatedness to any phage deposited in public databases, including *P. larvae* phages. From the 77 CDS predicted in the phage genome, 47 were confirmed by MS/MS. Genomic comparison analysis demonstrated that API480 is a singleton with 28 unique proteins, sharing only 19 of its proteins with other *P. larvae* siphoviruses, mostly with Harrison and Vegas clusters. This can be interpreted as a recent evolutionary history between API480 and these phages. It was surprising to see how the API480 host lysis proteins seem to be composed of one endolysin and two putative holins and how these proteins are the most conserved among *P. larvae* phages. Although these putative holins need to be experimentally confirmed, they apparently provide a new way for phages to lyse its host cells at the end of its lytic cycle. As the majority of phages known today, API480 and the other *P. larvae* phages also have their genomes organised in mosaics, starting with the structural and morphogenetic modules that are followed by the host lysis, DNA replication and metabolism related proteins. They all seem to have an overall common structure despite the limited shared gene content between them. This reinforces the existence of a common evolutionary ancestor among these phages. The low frequency of singletons found in *P. larvae* phages (from n = 49, 4% are singletons) which differs from other phage populations infecting hosts of similar taxonomic level, such as *Bacillus* (from n = 83, 18.1% are singletons)^12^ and *Gordonia* phages (from n = 79, 17.7% are singletons)^13^, indicates that the full genetic diversity is still untapped. This can be the result of similar isolation techniques used but not the outcome of low environmental sampling diversity, as *P. larvae* phages have been isolated from different isolation sources and geographical regions^11^. Overall, the genomic and proteomic analysis show that API480 is a completely distinct phage, suggesting the creation of a new genus within the *Podoviridae* family.

The analysis of the firstly reported genetic sequence of API480, widely distinct from the others (maximum genome coverage of 5%), encouraged its detailed characterization considering its use in AFB control.

The evaluation of API480 life cycle was accomplished based on the *in silico* analysis together with the assessment of the phage ability to integrate the bacterial host genome. The ability to lysogenise bacteria by a temperate phage is based on a gene cluster responsible for integration (integrase) and maintenance (repressors of the lytic cycle) of the prophage. Unlike all the other *P. larvae* phages no lysogeny module and no integrase gene were found in API480 genome.

The temperate nature of the phage was confirmed by the detection of a phage gene in the host genome and by the conversion of Pl02–27 into a phage-resistant strain. Although the use of temperate phages for therapeutic purposes is accepted, it raises several concerns^14^. Some strategies are known to safeguard its use for this purpose based on the difficulty of isolating strictly lytic phages, which was already reported for other bacterial species. Recently, Nale *et al*.^15^ revealed that all published *Clostridium difficile* phages are so far temperate and encode an integrase gene. Meader and colleagues (2010)^16^ anticipated that this is possibly due to the high incidence of prophage genes, revealing resistance to further infections. They also infer that the spore form may favour phage integration into the genome.

The use of optimized phage combinations with distinct and often complementary features (such as host range), as a strategy to lighten the effect of lysogeny and consequent phage resistance, has already been demonstrated^14^. The susceptibility of R-Pl27 strains to other *P. larvae* phages from our collection confirmed this idea.

Recent reports on the use of temperate phages for therapy were recently reviewed^17^. For example, phage ØCD27 was used to control *C. difficile* infections reducing their presence in the colon and decreasing the toxin expression^18^. Nale *et al*.^14^ reported the reduction of *C. difficile* colonisation by a cocktail of temperate phages extending the life expectancy of mice. A *Pseudomonas aeruginosa* systemic infection was treated in flies and in a murine animal model using two temperate phages (MP22 and D3112)^19^.

As far as AFB control is concerned, spore infected lab-reared larvae were also successfully treated with cocktails of *P. larvae* phages with a temperate lifestyle^8,9^.

API480 revealed to have broad lytic spectra, being active against 69% of the isolated field strains. All these strains belong to ERIC I, the far more diverse worldwide genotype^20^ and the one that causes higher morbidity in hives^21^. API480 also seems to be able to infect and propagate in ERIC II strains. Nevertheless, no evidence was found that the phage can be used to control infections caused by ERIC III and IV strains, as in this case the lysis occur without phage replication.

According to Yost *et al*.^9^ phages show host preference for the ERIC group from which they were isolated, and the preference for ERIC I was also observed in other *P. larvae* phages.

The high specificity that this phage revealed for *P. larvae* is particularly important as far as the first-instar larval commensal bacteria*, L. kunkeei* and *P. apium* alpha 2.2 are concerned, as it indicates that this phage would not have a harmful impact on their gut microbiota^22,23^. While studying the phage growth parameters, API480 revealed a slow adsorption to its host (in 35 min) as reported for example in the *C. difficile* phage CDHM1 (30 min)^24^ and a release of as few as three progeny viruses per infected cell. A burst of around eight phages per cell was recently reported in the *P. larvae* phage HB10c2^7^ and a burst of five and seven phages per cell was obtained in other two temperate phages isolated from *C. difficile*, φC2 and φC5, respectively^25^. The latter authors hypothesised that these result could be due to suboptimal growth conditions, explanation also given by Touchon *et al*.^26^ while studying life-history traits of temperate phages associated with lysogeny. According to a study carried out by Hadas *et al*.^27^ with T4 phage, parameters of phage development and cell lysis are dependent on bacterial growth rate. Correspondingly, slow adsorption rates, small burst size and high latent period were expected in a slow-grower bacterium such as *P. larvae*^28^.

The stability of API480 in simulated hive conditions was assessed to learn about its suitability in AFB biocontrol. API480 was very stable when exposed to high glucose concentrations (frequently used for feeding bees) and to pH values higher than 4.0 (often found in the hive).

Moreover, the phage infectivity was almost unaffected by larval fluids (0.4 Log PFU.mL^−1^ reduction) indicating that, at least for 24 hours, this is a favourable compartment for the host infection. Nevertheless, before reaching the larvae, phages will be mixed with RJ during the crop content regurgitation back to the mouth^29^ and they will be available in brood combs for larval consumption. According to our results, larvae have no more than 6 hours to ingest phages in a viable state. The decrease in phage viability at some point was an expected result, as Ribeiro *et al*.^30^ have recently reported. Some RJ elements may be responsible for this antiviral effect, such as proteinases^31^ and phenolic compounds^32^ that probably interact with phage structural proteins, contributing to their inactivation^33^.

In conclusion, despite being a temperate phage, the API480 broad lytic spectra, the specificity to *P. larvae* and the behaviour when challenged by simulated hive conditions encouraged its use for therapeutic purposes. Furthermore, the possibility of administering it together with other *P. larvae* phages which are able to infect API480 resistant strains mitigates the lysogenic nature of the phage.

## Materials and Methods

### Bacterial strains and cultivation conditions

In this study, 23 previously isolated *P. larvae* strains were used: 13 field strains (Pl01-(01, 03, 07, 07b2, 13, 14, 18); Pl02-(21, 23, 27, 30b, 31); Pl03–28)^34^; three strains originally isolated in Spain in 2016 (Guadalajara) (Pl02–86, 87, 89); seven reference strains: LMG 9820, CCUG 48972, CCUG 48973; LMG 15974 and LMG 16252 LMG 16247, LMG 16250.

These strains were cultivated in MYPGP agar (10 g.L^−1^ Mueller-Hinton Broth (Oxoid); 15 g.L^−1^ yeast extract (Oxoid); 3 g.L^−1^ de K_2_HPO_4_ (LabKem); 1 g.L^−1^ de Sodium-pyruvate (Fisher); 2% glucose (Ameresco) and 17 g.L^−1^ agar (VWR) and incubated at 37 °C under 5% CO_2_ overnight (O/N).

*P. larvae* isolation was performed as described by Genersch and Otten (2003)^35^, from brood samples collected in 132 hives spread over the Portuguese territory (29 with visible signs of infection and 103 apparently non-infected): larvae were emulsified in 500 µL sterile water, heated at 90 °C, 6 min and sewed in MYPGP agar. After incubation for 3 to 6 days at 37 °C, 5% CO_2_, single colonies were propagated in MYPGP agar and stored at −80 °C with 20% glycerol.

Non-*P. larvae* strains used to assess phage specificity for *P. larvae* included: *Paenibacillus polymyxa* (LMG 13294), *Paenibacillus alvei* (LMG 13253), *Lactobacillus pentosus* (DSM 20314), *Lactobacillus rhamnosus* (CECT 288), *Lactobacillus paracasei* (CECT 277), *Lactobacillus casei* (CECT 5275), *Lactobacillus acidophilus* (ATCC 4356), *Bacillus subtilis* (DSMZ 10), *Bacillus coagulans* (CECT 12), *Bacillus cereus* (CEB collection), *Bacillus circulans* (CEB collection), *Lactobacillus kunkeei* (LMG 18925) and *Parasaccharibacter apium* alpha 2.2 (strain C6)^22^.

All *Lactobacillus* spp were cultured in MRS broth (Frilabo) and MRS 15 g.L^−1^ agar (VWR). *Bacillus* spp, *P. polymyxa* and *P. alvei* were cultured in Nutrient broth ((5 g.L^−1^ Peptone (Amresco) and 3 g.L^−1^ Meat extract (Fluka Biochemika)) and Nutrient agar (15 g.L^−1^ agar). *P. apium* alpha 2.2 was cultured in Sabouraud dextrose broth (SDB) and sewed in SDB 15 g.L^−1^ agar (VWR).

The CCUG strains were obtained from the Culture Collection of the Goteborg University, LMG from the BCCM - Belgian Coordinated Collections of Microorganisms, DSMZ from the Deutsche Sammlung von Mikroorganismen und Zellkulturen GmbH and CECT from the Colección Española de Cultivos Tipo.

### 16S-PCR identification of *P. larvae* and rep-PCR analysis

The bacterial DNA was purified from bacterial suspensions using the Quick-DNA Fungal/Bacterial Miniprep Kit (Zymo) and amplified using Kapa*Taq* (Kapa Biosystems) according to the manufacturer’s instructions.

The PCR primer sequences and conditions used for *P. larvae* identification (Supplementary Table S1) were based on the *P. larvae* 16S rRNA gene^36^. Positive results revealed a 1,106 bp band in a 1% (w/v) agarose gel under UV light.

The enterobacterial repetitive intergenic consensus (ERIC) genotyping of the previously identified *P. larvae* was accomplished through genomic fingerprinting as reported in Genersch and Otten (2003)^35^ (primers and conditions are detailed in Supplementary Table S1). ERIC patterns were visualised in a 2% agarose gel under UV light. LMG 9820, CCUG 48972 LMG 15974 and LMG 16247 were used as standard for ERIC I, II, III and IV profiles, respectively.

### Bacteriophage isolation and production

Soil samples from hive surroundings were used for phage isolation. For that, samples were mixed with groups of five different bacterial strains pre-cultured O/N in MYPGP broth (37 °C, 5% CO_2_). After another O/N incubation, the supernatant was filtered-sterilized through 0.22 µm PES membranes (GE Healthcare) and 10 µL were spotted on the respective bacterial lawn (bellow designated as “spot test”). For lawns preparation 100 µl of the freshly grown strain with 3 mL 0.4% MYPGP agar and poured into agar plates. After O/N incubation at 37 °C, 5% CO_2_ bacterial inhibition zones were picked and propagated over host bacterial lawns^37^. After a subsequent incubation, phages were isolated from a single phage plaque. A volume of 2 mL SM buffer (5.8 g.L^−1^ NaCl (PanReac); 2 g.L^−1^ MgSO_4_.7H_2_O (VWR); 50 mL.L^−1^ 1 M Tris-HCl pH 7.5 (VWR)) was added to the plates. The floating liquid together with the soft-agar were collected, centrifuged (10 min, 9000 × *g*, 4 °C) and filtered-sterilized through 0.22 µm PES membranes. Phages were stored at 4 °C until use.

For phage propagation, 10 µL of the stored phage suspension were spread evenly on host bacterial lawns^38^. Plates were incubated O/N at 37 °C with 5% CO_2_ and treated as described above for phage isolation, until a filter-sterilized high-titre phage suspension around 10^8^ PFU.mL^−1^ (PFU: phage plaque count) was obtained (stored at 4 °C). The diameter of six individual phage plaques was registered using a SZ40 Zoom Stereo Microscope (Olympus).

### Lytic spectra determination and efficiency of plating

The lytic activity of the isolated phage vB_PlaP_API480 (API480) was tested against 68 *P. larvae* strains through spot test, as described above for phage isolation. The presence of bacterial inhibition areas was indicative of host susceptibility to the phage, and these strains were further used to assess efficiency of plating (EOP). A volume of 10 µL of serial phage dilutions was placed in each new bacterial lawn and the drop was allowed to drip along the agar surface to facilitate the phage plaque counting. The relative EOP was calculated by dividing the PFU.mL^−1^ of each susceptible strain by the titre for the relevant propagating host (Pl02–27)^39^. The EOP was scored as 0 (negative), 1 (<10%), 2 (10–100%), 3 (>100%) and LFW (Lysis from without) if phage plaques are only visible at the highest dilutions.

### Electron microscopy analysis

Phage particles were collected by centrifugation (1 h, 25,000 × *g*, 4 °C) in a Beckman J2–21 centrifuge with a JA18.1 fixed rotor. The sediment was washed twice in tap water prior to centrifugation as above. Phages were deposited on copper grids with a carbon-coated Formvar film grid, stained with 2% uranyl acetate (pH 4.0) and examined using Jeol JEM 1400 transmission electron microscope (Tokyo, Japan).

### DNA isolation, genome sequencing and annotation

*P. larvae* API480 phage genomic DNA was isolated using the phenol-chloroform-isoamyl alcohol method essentially as described elsewhere^39^. DNA samples were further used for library construction using the Illumina Nextera XT library preparation kit. The DNA libraries generated were sequenced in the lllumina MiSeq platform, using 250 bp paired-end sequencing reads. An automatic initial treatment was performed on raw sequence data, namely adapters and low quality bases trimming. Demultiplexed reads were *de novo* assembled into a single contig using Geneious R9 (Biomatters, Newark, NJ, USA).

The assembled genomes were scanned through MyRAST to search for coding regions^40^ and tRNAscan-SE to search for tRNAs^41^. To search for function, proteins were analysed through BLASTP^42^ and HHpred^43^ using an E-value cutoff of 1 × 10^–5^ to search for similarities. Identified proteins were also analysed with TMHMM^44^ and SignalP to predict transmembrane domains and signal peptide cleavage sites^45^. Putative promoter regions were checked using PromoterHunter from phiSITE^46^ and were further manually verified. ARNold was used to predict factor-independent terminators^47^ and the energy was calculated using Mfold^48^. The total genome or proteome were checked for antibiotic resistance genes through the ResFinder^49^ and the Resistance Gene Identifier (RGI) of CARD (The Comprehensive Antibiotic Resistance Database). The selected criteria were the display of results with perfect, strict and loose hits. For the search of toxins, the Toxin-Antitoxin Database (TADB) was used with the TAfinder tool^50^.

### Comparative genomic analysis

To determine the relationship of the *P. larvae* API480 podovirus within all *P. larvae* phages, all complete genomes sequences deposited at GenBank as of June 2019 (n = 48) were retrieved and analysed as previously described^51^. Briefly, the shared gene content was analysed with Phamerator^52^. This program allowed (1) the assignment of all *P. larvae* phage gene products into phams (proteins with related sequences) or orphams (i.e. unique proteins) using with kclust, an alignment-free algorithm; (2) the generation of pairwise comparison genomic maps; and (3) the identification of conserved domains in all proteins using the NCBI conserved domain database. The resulting protein repertoire relatedness was visualized with SplitsTree^53^. Phage membership was assigned based on shared gene content a metric recently used to assigned staphylococcal phages^51^, using a cut-off of 40% of shared genes (phams) to assign phages solely in one cluster.

### Protein identification by mass spectrometry (ESI-MS/MS)

Mass spectrometry was performed as described in Oliveira *et al*.^54^. Briefly, phage proteins were extracted from a phage stock, prepared as described above (>10^9^ PFU.mL^−1^) using chloroform:methanol (Acros Organics) (1:1:0.75 in volume). Proteins were subsequently separated by standard SDS-PAGE and stained by Gelcode™ Blue Safe Protein Stain (Thermo Scientific). Bands spanning the entire lane of the gel were cut out and subjected to trypsin digestion. The resulting peptides were then identified by ESI-MS/MS based on a database file containing all predicted phage proteins.

### Phage adsorption and One-step growth curve

An O/N grown Pl02–27 culture was harvested by centrifugation (10 min, 8000 × *g*, 4 °C) and re-suspended in fresh medium to obtain an optical density at 620 nm (OD_620_) of 0.3 (approximately 3 × 10^7^ CFU.mL^−1^). The phage was added to the obtained suspension of Pl02–27 with a multiplicity of infection (MOI) of 0.1 and incubated at 37 °C with shaking (120 rpm in a PSU-10i Orbital Shaker, BIOSAN).

To assess the time the phage takes to adsorb to the host, samples of 50 µL were collected every 10 min and for 60 min and immediately chloroform-treated (1:10 (v/v)) and centrifuged (2 min, 8000 × *g*). The upper phase was serial diluted and the phage was titred (PFU.mL^−1^) in order to obtain the number of free phages^38^.

For the one-step growth curve (OSGC) the phage-host adsorption occurred for 35 min (as indicated by the adsorption assay) at 37 °C, 120 rpm. The mixture was then centrifuged (10 min, 8000 × *g*, 4 °C) and the pellet re-suspended in 10 mL fresh MYPGP broth medium. Samples were taken every 15 min over a period of 60 min and the phage titre was assessed.

The PFU.cell^−1^ was obtained through the ratio between the PFU.mL^−1^ in each time point and the initial PFU.mL^−1^. The burst size was estimated from the resultant sigmoid curve.

### Phage life cycle

The evaluation of the phage ability to integrate the bacterial host genome was adapted from a procedure suggested by Kalatzis *et al*.^55^. Briefly, an O/N grown culture of the strain Pl02–27 was harvested by centrifugation (10 min, 8,000 × *g*, 4 °C) and re-suspended in fresh medium to reach an OD_620_ of 0.3. After a tenfold dilution the host suspension was mixed with the phage to get a MOI of 50 and incubated at 37 °C, 5% CO_2_ for 24 hours with agitation (120 rpm).

Samples (n = 3) were serial diluted and poured in MYPGP agar (incubation at 37 °C, 5% CO_2_, O/N). About 10 bacterial colonies were isolated, cultured in solid media and re-cultured again in three serial passages.

The life cycle of API480 was assessed by investigating the presence of the phage into the host genome in each of the isolated colonies. The original Pl02–27 strain was used as control. For that, a specific primer pair for API480 was designed based on the CDS_12: 480_12 Fw 5′-CAGGAACTCAGACCCTACGC -3′ and 480_12 Rev 5′-GCCTGCTGCAAAGTCATACA-3′. A colony PCR reaction was performed in a MJ Mini Personal Thermal Cycler: 10 min at 95 °C followed by 30 cycles of 15 sec at 95 °C; 15 sec at 60 °C and 15 sec at 72 °C and a final extension of 3 min at 72 °C. For the reaction mixture a 1x Xpert Fast Master mix (Grisp) and 0.8 µM of each primer were used. A positive reaction revealed a 227 bp band in a 1% agarose gel.

The API480 activity against the obtained colonies was then investigated trough phage spot test in the respective bacterial lawn. Four Pl02-H27 bacterial clones newly resistant to the phage (designated as R-Pl27) were then tested for phage infection using other *P. larvae* bacteriophages isolated in previous works: phiIBB_Pl23^56^; vB_Pl_CEB16, vB_PlCEB_46, vB_PlCEB_51 and vB_PlCEB_55 (unpublished phages from CEB collection).

### Phage specificity and stability in simulated field conditions

The phage specificity for *P. larvae* was evaluated trough spot test on lawns of the above-mentioned non-*P. larvae* bacterial strains including the first instars larval commensal strains *L. kunkeei* and *P. apium* alpha 2.2 (strain C6)^22^.

API480 stability in simulated hive products/solutions was assessed for 24 hours: phage (final concentration of 10^7^ PFU.mL^−1^) was incubated in a 50% (w/v) sucrose solution at room temperature (envisaging phage administration in bees’s artificially feed), in royal jelly (RJ) at 37 °C, 5% CO_2_ (where first–instars larvae lay on) and in solutions with acidic pH values at 37 °C, 5% CO_2_ (that include those occurring in the hive^57,58^).

For the assay with RJ (supplied by Apiguarda, Portugal), API480 was 1:10 (v/v) diluted in 100 µL of this hive-product and incubated for 0, 1, 3, 6, 8 and 24 hours (3 reaction tubes were prepared for each time point). After the addition of 900 µL SM buffer the mixture was homogenised by vortexing and 200 µL of chloroform were added. Tubes were vortexed and centrifuged (2 min, 14000 × *g*) and the upper phase was titred (PFU.mL^−1^).

For the pH assay distinct solutions of universal buffer (UB) (150 mM KCl (PanReac), 10 mM KH_2_PO_4_ (PanReac), 10 mM Sodium-Citrate (Thermo Fisher Scientific) and 10 mM H_3_BO_3_ (Thermo Fisher Scientific), were adjusted with HCl (Acros Organics) to obtain a pH range between 3.0 to 5.0 with intervals of 0.5 units. API480 was 1:10 (v/v) diluted and incubated. Two controls were used in this experiment: phage in SM buffer (pH 7.4) and phage in UB (pH 7.4). Phage concentration (PFU.mL^−1^) was assessed at 0, 1, 3, 6, 8 and 24 hours.

The phage infectivity was still performed in 15 healthy larvae gathered from larvae combs, that were weighted, individually homogenised and incubated with API480 for 24 hours: 100 µL of each homogenate with 10^7^ PFU.mL^−1^.

After, 900 µL of SM buffer and 200 µL of chloroform were added. Phages were collected from the upper phase of the mixture after centrifugation (2 min, 14000 × *g*) and titred (PFU.mL^−1^).

### Statistical analysis

The statistical analysis of the results was performed using GraphPad Prism 7. In all the assays, means and standard deviations were determined based on 3 independent experiments (n = 3). Results were compared using t-test (phage stability in sucrose 50% (w/v)), one-way ANOVA, with Turkey’s multiple comparison statistical test (phage viability on RJ, phage adsorption and OSGC), and two-way ANOVA, with Turkey’s multiple comparison statistical test (phage stability with acidic pH and in larvae homogenised). All tests were performed with a confidence level of 95%. Differences were considered statistically different if p ≤ 0.05.

## Supplementary information

Supplementary Information.
