# Supplementary material for: Characterization of a new podovirus infecting Paenibacillus larvae
Source: Sci Rep. 2019 Dec 30;9:20355. doi: 10.1038/s41598-019-56699-y (PMC6937236; doi:10.1038/s41598-019-56699-y)
Supplement: Supplementary file 1 — Supplementary Information. [file 41598_2019_56699_MOESM1_ESM.pdf]

Supplementary Information

Characterization of a new podovirus infecting *Paenibacillus larvae*

Henrique G. Ribeiro[1], Luís D. R. Melo[1], Hugo Oliveira[1], Maarten Boon[2], Rob Lavigne[2], Jean-Paul Noben[3], Joana Azeredo[1] and Ana Oliveira*[1]

[1]CEB - Centre of Biological Engineering, LIBRO - Laboratório de Investigação em Biofilmes Rosário Oliveira, University of Minho, 4710-057, Braga, Portugal.
[2]Laboratory of Gene Technology, KU Leuven, Leuven, Belgium.
[3]Biomedical Research Institute and Transnational University Limburg, Hasselt University, Agoralaan D, 3590 Hasselt, Belgium
*Corresponding Author: anaoliveira@deb.uminho.pt

Table S1: PCR conditions for 16S-PCR of *P. larvae* identification and rep-PCR genotyping.

Target gene	Sequence (5′-3′)	PCR conditions
**16S rRNA**	Fw: CTTGTGTTTCTTTCGGGAGACGCC<br>Rev: TCTTAGAGTGCCCACCTCTGCG	10 min 95 °C<br>15 sec 95 °C; 15 sec 60 °C; 15 sec 72 °C (30 cycles)<br>3 min 72 °C
**rep-PCR**	ERIC2: AAGTAAGTGACTGGGGTGAGC<br>ERIC1R: ATGTAAGCTCCTGGGGATTCA	3 min 95 °C<br>45 sec 95 °C; 30 sec 45 °C; 3 min 72 °C (30 cycles)<br>10 min 72 °C

Table S2: API480 genome annotation and BLASTP or HHpred homology. CDSs unique to API480 are in white; CDSs homologue to bacteria are coloured in light grey; CDSs homologue to phages are coloured in dark grey; proteins with an identified function are highlighted in bold. [1] indicates proteins with assigned function validated by ESI-MS/MS.

gp	Ni	Nf	NN	+/-	AA	Product	Blastp/HHpred	E-value
1	1	426	426	+	141	Hypothetical protein	Hypothetical protein [Bacillus thuringiensis]	3E-13
2	404	658	255	+	84	Hypothetical protein		
3	655	963	309	+	102	Hypothetical protein	Hypothetical protein [Bacillus phage Mgbh1]	6E-18
4	956	2407	1452	+	483	**Terminase large subunit [1]**	Phage terminase, large subunit, PBSX family (TIGR01547) [uncultured Mediterranean phage uvMED]	2E-43
5	2420	2611	192	+	63	Hypothetical protein		
6	2627	4405	1779	+	592	**Portal protein [1]**	Portal protein [Enterobacteria phage P22]	1E-46
7	4562	5371	810	+	269	Hypothetical protein	Hypothetical protein [Bacillus cereus]	1E-21
8	5462	6403	942	+	313	**Major capsid protein [1]**	Major capsid protein [Bacillus phage BM5]	1E-39
9	6470	6733	264	+	87	Hypothetical protein	Hypothetical protein [Bacillus thuringiensis]	1E-15
10	6772	7152	381	+	126	Hypothetical protein	Hypothetical protein [Bacillus sp. J37]	2E-06
11	7161	9152	1992	+	663	Hypothetical protein		
12	9164	10342	1179	+	392	Hypothetical protein		
13	10354	10647	294	+	97	Hypothetical protein		
14	10649	11578	930	+	309	Hypothetical protein		
15	11591	12559	969	+	322	Hypothetical protein		
16	12564	17459	4896	+	1631	Hypothetical protein		
17	17841	18083	243	+	80	**Holin [1]**	Holin_BhlA [PF10960.8 hit]	8E-23
18	18101	18772	672	+	223	**Endolysin**	N-acetylmuramoyl-L-alanine amidase [Paenibacillus phage Vegas]	7E-159
19	18778	19248	471	+	156	Hypothetical protein	Hypothetical protein [Paenibacillus larvae]	2E-109
20	19266	19541	276	+	91	Hypothetical protein	Hypothetical protein WANDERER_22 [Paenibacillus phage Wanderer]	9E-53
21	19554	19793	240	+	79	**Holin [1]**	Phage_holin_5_1 [PF06946.11 hit]	2E-17
22	19881	20150	270	-	89	Hypothetical protein		
23	20190	20432	243	-	80	Hypothetical protein	Nucleotide binding domain [Escherichia coli K12]	1E-12
24	20425	21408	984	-	327	**DNA-binding protein [1]**	ParM/StbA family protein [Bacillus mycoides]	3E-59
25	21603	21893	291	-	96	Hypothetical membrane protein	Hypothetical protein [Paenibacillus larvae]	7E-47

26	21920	22249	330	-	109	**mRNA-degrading endonucleas** [1]	mRNA-degrading endonuclease [Paenibacillus larvae]	2E-73
27	22246	22515	270	-	89	**Antitoxin MazE**	Antitoxin MazE [Paenibacillus larvae subsp. larvae]	3E-54
28	22580	22777	198	+	65	Hypothetical membrane protein		
29	22906	23064	159	-	52	Hypothetical protein		
30	23057	23431	375	-	124	Hypothetical protein	Hypothetical protein [Paenibacillus larvae]	6E-75
31	23415	23603	189	-	62	Hypothetical protein	Hypothetical protein [Paenibacillus larvae]	4E-31
32	23600	23800	201	-	66	Hypothetical membrane protein		
33	23805	23996	192	-	63	Hypothetical membrane protein		
34	23981	24241	261	-	86	Hypothetical protein	Hypothetical protein TRIPP_91 [Paenibacillus phage Tripp]	2E-37
35	24243	24428	186	-	61	Hypothetical membrane protein		
36	24484	24864	381	-	126	Hypothetical protein	Hypothetical protein [Paenibacillus larvae]	3E-71
37	24871	25161	291	-	96	Hypothetical protein	Hypothetical protein WANDERER_61 [Paenibacillus phage Wanderer]	8E-60
38	25154	25477	324	-	107	Hypothetical protein	Hypothetical protein C7CDELTA_68 [Paenibacillus phage C7Cdelta]	6E-58
39	25507	26010	504	-	167	**dUTP pyrophosphatase** [1]	dUTP pyrophosphatase [Paenibacillus phage C7Cdelta]	9E-72
40	26022	26282	261	-	86	Hypothetical protein	Hypothetical protein VEGAS_60 [Paenibacillus phage Vegas]	7E-12
41	26298	26417	120	-	39	Hypothetical protein	Hypothetical protein VEGAS_59 [Paenibacillus phage Vegas]	3E-20
42	26414	26602	189	-	62	Hypothetical protein	Hypothetical protein [Paenibacillus larvae]	1E-22
43	26605	26805	201	-	66	Hypothetical protein	Hypothetical protein [Paenibacillus larvae]	4E-34
44	26798	27301	504	-	167	**Resolvase** [1]	Holliday junction resolvase [Escherichia coli]	2,80E-24
45	27303	27497	195	-	64	Hypothetical protein		
46	27613	29838	2226	-	741	**DNA polymerase I** [1]	DNA polymerase I [Geobacillus stearothermophilus]	3E-76
47	29870	32662	2793	-	930	**DNA primase** [1]	DNA primase small subunit [Bacillus phage vB_BboS-125]	2E-12
48	32686	33147	462	-	153	**Single-stranded DNA-binding protein** [1]	Single-stranded DNA-binding protein [Paenibacillus larvae]	9E-86
49	33214	33756	543	-	180	Hypothetical protein		
50	33807	34355	549	-	182	**Host-nuclease inhibitor** [1]	Host-nuclease inhibitor [Desulfovibrio vulgaris]	3E-28
51	34336	34542	207	-	68	Hypothetical protein	Hypothetical protein WANDERER_58 [Paenibacillus phage Wanderer]	3E-17
52	34532	34774	243	-	80	Hypothetical protein	Hypothetical protein [Paenibacillus larvae]	1E-49
53	34767	34997	231	-	76	Hypothetical protein	Hypothetical protein [Paenibacillus larvae]	3E-38
54	35004	35312	309	-	107	Hypothetical protein	Hypothetical protein [Paenibacillus larvae]	6E-69
55	35359	35541	183	-	60	Hypothetical protein	Hypothetical protein WANDERER_53 [Paenibacillus phage Wanderer]	7E-26

56	35538	35798	261	-	86	Hypothetical protein		
57	35802	37370	1569	-	522	**DEAD/DEAH box helicase** [1]	DEAD/DEAH box helicase [Bacillus thuringiensis]	3E-129
58	37363	37515	153	-	50	Hypothetical protein		
59	37796	38482	687	-	228	Hypothetical protein	Hypothetical protein [Bacillus cereus]	8E-44
60	38495	39013	519	-	172	Hypothetical protein	Hypothetical protein [Bacillus cereus]	2E-20
61	39031	39198	168	-	55	Hypothetical membrane protein		
62	39188	39346	159	-	52	Hypothetical protein		
63	39387	39638	252	-	83	Hypothetical protein	Hypothetical protein [Paenibacillus larvae]	2E-43
64	39673	39876	204	-	67	Hypothetical protein		
65	39889	40290	402	-	133	Hypothetical protein		
66	40355	40981	627	-	208	Hypothetical protein		
67	40968	41144	177	-	58	Hypothetical membrane protein		
68	41189	41380	192	-	63	Hypothetical protein		
69	41402	41584	183	-	60	Hypothetical protein		
70	41614	42114	501	-	166	**Antirestriction protein ArdA** [1]	Antirestriction protein ArdA [Paenibacillus larvae]	6E-26
71	42165	42347	183	-	60	Hypothetical protein	Hypothetical protein [Paenibacillus larvae]	5E-24
72	42358	42618	261	-	86	Hypothetical protein		
73	42742	42987	246	-	81	Hypothetical protein	Hypothetical protein [Brevibacillus laterosporus]	1E-22
74	42992	43219	228	-	75	Hypothetical protein	Hypothetical protein [Paenibacillus larvae]	1E-36
75	43546	43677	132	-	43	Hypothetical protein		
76	43704	44159	456	-	151	Hypothetical protein	Hypothetical protein [Paenibacillus larvae]	1E-32
77	44461	44676	216	-	71	Hypothetical protein	Hypothetical protein JENST_60 [Brevibacillus phage Jenst]	5E-07

Table S3: Shared gene content. The complete genome sequences of all *P. larvae* phages (n=49) was comparative analysed through Phamerator and the resulting shared gene content matrix was plotted. Phamerator have assigned gene products into phams (proteins with related sequences) or orphams (i.e. unique proteins). Phage groups are defined into cluster (phage groups) on the basis of sharing > 40% of their proteins with all members or as or singletons (single phages) if sharing fewer proteins. In the upper part, the cluster division is highlighted with colours and match the previous classification here (API480 in red). Phages that share > 40% of their gene products are coloured in grey.

	FERN																														HARRISON		VEGAS							LILY	HALCYONE								API140
PAGASSA	100	97.1	95.7	93.4	83.8	83.8	79.8	79.8	88	87	68.9	70.1	70.1	72.6	67	61.2	61.2	61.2	61.2	61.2	61.2	69.3	60.8	62.8	66.2	73.9	66.2	62.6	61.6	61.5	29	29	31.3	31.3	31.3	31.7	17.5	21.6	21.6	28	1.3	1.3	1.3	2.6	2.6	2.6	1.4	2.6	4.1
HONEYBEAR	97.1	100	98.5	89.9	83.4	83.4	82.2	82.2	86.4	89.6	70.9	72.1	72.1	74.8	68.8	63.1	63.1	63.1	63.1	63.1	63.1	71.3	62.6	64.7	68.3	76	68.2	63.2	62.1	62.1	30	30	32.4	32.4	32.4	32.7	16.5	22.3	22.3	28.9	1.4	1.4	1.4	2.7	2.7	2.7	1.4	2.6	4.3
TOOTHLESS	95.7	98.5	100	91.3	84.7	84.7	83.5	83.5	87.8	91.1	72	73.2	73.2	76	69.8	64.1	64.1	64.1	64.1	64.1	64.1	72.4	63.6	65.7	69.5	77.1	69.3	64.3	63.2	63.1	30.6	30.6	30.3	30.3	30.3	30.6	16.8	22.6	22.6	29.4	1.4	1.4	1.4	2.7	2.7	2.7	1.5	2.7	4.3
TADHANA	93.4	89.9	91.3	100	90.1	90.1	81.2	81.2	84.1	82.8	69.9	71	71	67.7	69.3	65.1	65.1	65.1	65.1	65.1	65.1	75	66.2	68.2	67.4	76.5	67.2	63.7	62.6	62.6	33	33	30	30	30	30.3	18.2	22.4	22.4	30.6	2.7	2.7	2.7	4	4	4	2.9	4	4.3
FERN	83.8	83.4	84.7	90.1	100	100	90.1	90.1	74.9	83.8	63.7	64.8	64.8	72.2	64.6	60.6	60.6	60.6	60.6	60.6	60.6	70.3	70.7	72.6	61.5	70.1	62.7	60.8	58.3	69.4	32.2	32.2	30.5	30.5	30.5	30.9	19.3	23.4	23.4	29.9	5.3	5.3	5.3	3.9	3.9	3.9	2.8	3.9	5.6
WILLOW	83.8	83.4	84.7	90.1	100	100	90.1	90.1	74.9	83.8	63.7	64.8	64.8	72.2	64.6	60.6	60.6	60.6	60.6	60.6	60.6	70.3	70.7	72.6	61.5	70.1	62.7	60.8	58.3	69.4	32.2	32.2	30.5	30.5	30.5	30.9	19.3	23.4	23.4	29.9	5.3	5.3	5.3	3.9	3.9	3.9	2.8	3.9	5.6
LUCIELLE	79.8	82.2	83.5	81.2	90.1	90.1	100	100	72.3	82.8	73	74.3	74.3	70.8	67.6	60.5	60.5	60.5	60.5	60.5	60.5	64.1	67.7	66.7	70.4	71.7	71.8	68.1	65.6	76.8	26.2	26.2	30	30	30	30.3	16.7	22.4	22.4	29.1	4.1	4.1	4.1	2.7	2.7	2.7	1.4	2.7	5.7
SAUDAGE	79.8	82.2	83.5	81.2	90.1	90.1	100	100	72.3	82.8	73	74.3	74.3	70.8	67.6	60.5	60.5	60.5	60.5	60.5	60.5	64.1	67.7	66.7	70.4	71.7	71.8	68.1	65.6	76.8	26.2	26.2	30	30	30	30.3	16.7	22.4	22.4	29.1	4.1	4.1	4.1	2.7	2.7	2.7	1.4	2.7	5.7
BN12	88	86.4	87.8	84.1	74.9	74.9	72.3	72.3	100	79.5	67.5	68.8	68.8	71.1	65.7	60	60	60	60	60	60	64.9	59.5	61.5	62.1	69.4	62	57.2	57.5	56.1	28.4	28.4	29.3	29.3	29.3	27.1	15.7	21.1	21.1	28.8	1.3	1.3	1.3	5.1	5.1	5	1.4	2.5	4
KAWIKA	87	89.6	91.1	82.8	83.8	83.8	82.8	82.8	79.5	100	64.3	65.4	65.4	74.1	63.7	59.8	59.8	59.8	59.8	59.8	59.8	67.8	62.3	64.2	63.4	69.2	64.7	61.2	58.8	61.5	30.4	30.4	31.3	31.3	31.3	31.7	17.5	28.8	28.8	29.4	2.6	2.6	2.6	2.6	2.6	2.6	1.4	2.6	8.2
KIEL007	68.9	70.9	72	69.9	63.7	63.7	73	73	67.5	64.3	100	98.4	98.4	84.5	89.2	66.2	66.2	66.2	66.2	66.2	66.2	66.7	62.6	63.2	82	72.8	71.4	66.3	81.5	68	28	28	26.4	26.4	26.4	26.6	17	21.3	21.3	29.6	4.2	4.2	4.2	2.7	2.7	2.7	1.5	4.1	4.4
REDBUD	70.1	72.1	73.2	71	64.8	64.8	74.3	74.3	68.8	65.4	98.4	100	100	84.3	90.6	67.3	67.3	67.3	67.3	67.3	67.3	67.8	63.6	64.3	83.5	74	72.7	67.5	80	67.8	28.6	28.6	26.9	26.9	26.9	27.1	17.3	21.7	21.7	30.2	4.2	4.2	4.3	2.8	2.8	2.8	1.5	4.2	4.4
RANI	70.1	72.1	73.2	71	64.8	64.8	74.3	74.3	68.8	65.4	98.4	100	100	84.3	90.6	67.3	67.3	67.3	67.3	67.3	67.3	67.8	63.6	64.3	83.5	74	72.7	67.5	80	67.8	28.6	28.6	26.9	26.9	26.9	27.1	17.3	21.7	21.7	30.2	4.2	4.2	4.3	2.8	2.8	2.8	1.5	4.2	4.4
ELTIGRE	72.6	74.8	76	67.7	72.2	72.2	70.8	70.8	71.1	74.1	84.5	84.3	84.3	100	81.7	61.1	61.1	61.1	61.1	61.1	61.1	66.2	69.7	73.2	69.2	65.8	60.2	57	68.8	64.3	32.5	32.5	28.1	28.1	28.1	28.4	17.9	22.1	22.1	28.7	5.4	5.4	5.4	2.7	2.7	2.6	1.4	3.9	4.2
HB10C2	67	68.8	69.8	69.3	64.6	64.6	67.6	67.6	65.7	63.7	89.2	90.6	90.6	81.7	100	60.4	60.4	60.4	60.4	60.4	60.4	65.9	61.7	64.1	76.5	70.3	64.6	61.5	72.9	60.5	31.6	31.6	28.3	28.3	28.3	28.6	18.1	22.7	22.7	28.5	4.5	4.5	4.5	3	3	2.9	1.6	4.4	4.7
ARCTICFREEZE	61.2	63.1	64.1	65.1	60.6	60.6	60.5	60.5	60	59.8	66.2	67.3	67.3	61.1	60.4	100	98.5	98.5	98.5	98.5	98.5	66.7	62.6	75.2	61.1	63.1	63.7	58.9	62.1	55	32.7	32.7	28.4	28.4	28.4	28.7	27.1	19.3	19.3	39	2.7	2.7	2.7	2.7	2.7	2.7	1.4	1.3	5.7
DEVRI	61.2	63.1	64.1	65.1	60.6	60.6	60.5	60.5	60	59.8	66.2	67.3	67.3	61.1	60.4	98.5	100	98.5	98.5	98.5	98.5	66.7	62.6	75.2	61.1	63.1	63.7	58.9	62.1	55	32.7	32.7	28.4	28.4	28.4	28.7	27.1	19.3	19.3	39	2.7	2.7	2.7	2.7	2.7	2.7	1.4	1.3	5.7
BLOOM	61.2	63.1	64.1	65.1	60.6	60.6	60.5	60.5	60	59.8	66.2	67.3	67.3	61.1	60.4	98.5	98.5	100	98.5	98.5	98.5	66.7	62.6	75.2	61.1	63.1	63.7	58.9	62.1	55	32.7	32.7	28.4	28.4	28.4	28.7	27.1	19.3	19.3	39	2.7	2.7	2.7	2.7	2.7	2.7	1.4	1.3	5.7
JACOPO	61.2	63.1	64.1	65.1	60.6	60.6	60.5	60.5	60	59.8	66.2	67.3	67.3	61.1	60.4	98.5	98.5	98.5	100	98.5	98.5	66.7	62.6	75.2	61.1	63.1	63.7	58.9	62.1	55	32.7	32.7	28.4	28.4	28.4	28.7	27.1	19.3	19.3	39	2.7	2.7	2.7	2.7	2.7	2.7	1.4	1.3	5.7
GENKI	61.2	63.1	64.1	65.1	60.6	60.6	60.5	60.5	60	59.8	66.2	67.3	67.3	61.1	60.4	98.5	98.5	98.5	98.5	100	98.5	66.7	62.6	75.2	61.1	63.1	63.7	58.9	62.1	55	32.7	32.7	28.4	28.4	28.4	28.7	27.1	19.3	19.3	39	2.7	2.7	2.7	2.7	2.7	2.7	1.4	1.3	5.7
GRYPHONIAN	61.2	63.1	64.1	65.1	60.6	60.6	60.5	60.5	60	59.8	66.2	67.3	67.3	61.1	60.4	98.5	98.5	98.5	98.5	98.5	100	66.7	62.6	75.2	61.1	63.1	63.7	58.9	62.1	55	32.7	32.7	28.4	28.4	28.4	28.7	27.1	19.3	19.3	39	2.7	2.7	2.7	2.7	2.7	2.7	1.4	1.3	5.7
LIKHA	69.3	71.3	72.4	75	70.3	70.3	64.1	64.1	64.9	67.8	66.7	67.8	67.8	66.2	65.9	66.7	66.7	66.7	66.7	66.7	66.7	100	64.6	65.2	63	68.4	71.8	68.1	69.9	62.6	37.2	37.2	30	30	30	30.3	16.7	22.4	22.4	36.4	4.1	4.1	4.1	4	4	4	2.9	6.7	5.7
PHIIBB_PL23	60.8	62.6	63.6	66.2	70.7	70.7	67.7	67.7	59.5	62.3	62.6	63.6	63.6	69.7	61.7	62.6	62.6	62.6	62.6	62.6	62.6	64.6	100	74.6	57.7	62.6	58.6	55.5	55.9	60.1	43.3	43.3	28.1	28.1	28.1	28.4	16.4	22.1	22.1	31.5	4	4	4	2.7	2.7	2.6	2.8	5.3	7
YERFFEJ	62.8	64.7	65.7	68.2	72.6	72.6	66.7	66.7	61.5	64.2	63.2	64.3	64.3	73.2	64.1	75.2	75.2	75.2	75.2	75.2	75.2	65.2	74.6	100	58.2	63.3	56.3	53.3	55	57.8	39.9	39.9	29	29	29	29.3	22.1	18.8	18.8	31	5.3	5.3	5.3	3.9	3.9	3.9	2.8	3.9	4.2
SITARA	66.2	68.3	69.5	67.4	61.5	61.5	70.4	70.4	62.1	63.4	82	83.5	83.5	69.2	76.5	61.1	61.1	61.1	61.1	61.1	61.1	63	57.7	58.2	100	85.8	84.4	79.7	84.4	74.3	24.3	24.3	29.1	29.1	29.1	29.4	17	18.2	18.2	28.6	5.1	5.1	5.1	3.8	3.8	3.7	1.3	5	4
DIVA	73.9	76	77.1	76.5	70.1	70.1	71.7	71.7	69.4	69.2	72.8	74	74	65.8	70.3	63.1	63.1	63.1	63.1	63.1	63.1	68.4	62.6	63.3	85.8	100	89.2	83.3	76.2	77.4	27.4	27.4	30	30	30	30.3	17.4	20.3	20.3	32	1.4	1.4	1.4	1.4	1.4	1.4	1.5	2.8	4.5
SHELLY	66.2	68.2	69.3	67.2	62.7	62.7	71.8	71.8	62	64.7	71.4	72.7	72.7	60.2	64.6	63.7	63.7	63.7	63.7	63.7	63.7	71.8	58.6	56.3	84.4	89.2	100	93.3	85.3	84.6	28.2	28.2	30.5	30.5	30.5	30.9	16.3	20.4	20.4	37	2.7	2.7	2.7	2.6	2.6	2.6	1.4	5.2	5.6
XENIA	62.6	63.2	64.3	63.7	60.8	60.8	68.1	68.1	57.2	61.2	66.3	67.5	67.5	57	61.5	58.9	58.9	58.9	58.9	58.9	58.9	68.1	55.5	53.3	79.7	83.3	93.3	100	84.6	85	26.5	26.5	28.7	28.7	28.7	29	16.8	19.3	19.3	34.9	2.5	2.5	2.5	2.5	2.5	2.5	1.3	4.9	5.3
LEYRA	61.6	62.1	63.2	62.6	58.3	58.3	65.6	65.6	57.5	58.8	81.5	80	80	68.8	72.9	62.1	62.1	62.1	62.1	62.1	62.1	69.9	55.9	55	84.4	76.2	85.3	84.6	100	73.7	26.7	26.7	27.7	27.7	27.7	28	18.3	18.1	18.1	32.4	5	5	5.1	3.7	3.7	3.7	1.3	6.2	5.3
PBL1C	61.5	62.1	63.1	62.6	69.4	69.4	76.8	76.8	56.1	61.5	68	67.8	67.8	64.3	60.5	55	55	55	55	55	55	62.6	60.1	57.8	74.3	77.4	84.6	85	73.7	100	24.7	24.7	29.3	29.3	29.3	29.7	17.9	20.3	20.3	34.2	4.9	4.9	4.9	2.4	2.4	2.4	1.3	4.8	5.2
HARRISON	29	30	30.6	33	32.2	32.2	26.2	26.2	28.4	30.4	28	28.6	28.6	32.5	31.6	32.7	32.7	32.7	32.7	32.7	32.7	37.2	43.3	39.9	24.3	27.4	28.2	26.5	26.7	24.7	100	98.8	34.1	34.1	34.1	34.5	25.3	28.8	28.8	17.8	1.2	1.2	1.2	1.2	1.2	1.2	1.3	4.6	11.2
PAISLEY	29	30	30.6	33	32.2	32.2	26.2	26.2	28.4	30.4	28	28.6	28.6	32.5	31.6	32.7	32.7	32.7	32.7	32.7	32.7	37.2	43.3	39.9	24.3	27.4	28.2	26.5	26.7	24.7	98.8	100	34.1	34.1	34.1	34.5	25.3	28.8	28.8	17.8	1.2	1.2	1.2	1.2	1.2	1.2	1.3	4.6	11.2
DIANE	31.3	32.4	30.3	30	30.5	30.5	30	30	29.3	31.3	26.4	26.9	26.9	28.1	28.3	28.4	28.4	28.4	28.4	28.4	28.4	30	28.1	29	29.1	30	30.5	28.7	27.7	29.3	34.1	34.1	100	100	100	98.8	50	41.5	41.5	12.6	2.3	2.3	2.3	3.4	3.4	2.3	0	2.3	9.8
VADIM	31.3	32.4	30.3	30	30.5	30.5	30	30	29.3	31.3	26.4	26.9	26.9	28.1	28.3	28.4	28.4	28.4	28.4	28.4	28.4	30	28.1	29	29.1	30	30.5	28.7	27.7	29.3	34.1	34.1	100	100	100	98.8	50	41.5	41.5	12.6	2.3	2.3	2.3	3.4	3.4	2.3	0	2.3	9.8
VEGAS	31.3	32.4	30.3	30	30.5	30.5	30	30	29.3	31.3	26.4	26.9	26.9	28.1	28.3	28.4	28.4	28.4	28.4	28.4	28.4	30	28.1	29	29.1	30	30.5	28.7	27.7	29.3	34.1	34.1	100	100	100	98.8	50	41.5	41.5	12.6	2.3	2.3	2.3	3.4	3.4	2.3	0	2.3	9.8
HAYLEY	31.7	32.7	30.6	30.3	30.9	30.9	30.3	30.3	27.1	31.7	26.6	27.1	27.1	28.4	28.6	28.7	28.7	28.7	28.7	28.7	28.7	30.3	28.4	29.3	29.4	30.3	30.9	29	28	29.7	34.5	34.5	98.8	98.8	98.8	100	50.6	41.9	41.9	12.7	2.4	2.4	2.4	3.5	3.5	2.3	0	2.3	10
DRAGOLIR	17.5	16.5	16.8	18.2	19.3	19.3	16.7	16.7	15.7	17.5	17	17.3	17.3	17.9	18.1	27.1	27.1	27.1	27.1	27.1	27.1	16.7	16.4	22.1	17	17.4	16.3	16.8	18.3	17.9	25.3	25.3	50	50	50	50.6	100	46.4	46.4	15.5	2.6	2.6	2.6	1.3	1.3	2.6	0	1.3	5.5
LINCOLNB	21.6	22.3	22.6	22.4	23.4	23.4	22.4	22.4	21.1	28.8	21.3	21.7	21.7	22.1	22.7	19.3	19.3	19.3	19.3	19.3	19.3	22.4	22.1	18.8	18.2	20.3	20.4	19.3	18.1	20.3	28.8	28.8	41.5	41.5	41.5	41.9	46.4	100	100	13.9	2.6	2.6	2.6	1.3	1.3	1.3	1.4	2.5	13.6
WANDERER	21.6	22.3	22.6	22.4	23.4	23.4	22.4	22.4	21.1	28.8	21.3	21.7	21.7	22.1	22.7	19.3	19.3	19.3	19.3	19.3	19.3	22.4	22.1	18.8	18.2	20.3	20.4	19.3	18.1	20.3	28.8	28.8	41.5	41.5	41.5	41.9	46.4	100	100	13.9	2.6	2.6	2.6	1.3	1.3	1.3	1.4	2.5	13.6
LILY	28	28.9	29.4	30.6	29.9	29.9	29.1	29.1	28.8	29.4	29.6	30.2	30.2	28.7	28.5	39	39	39	39	39	39	36.4	31.5	31	28.6	32	37	34.9	32.4	34.2	17.8	17.8	12.6	12.6	12.6	12.7	15.5	13.9	13.9	100	1.3	1.3	1.3	1.2	1.2	2.5	0	3.7	5.3
ASH	1.3	1.4	1.4	2.7	5.3	5.3	4.1	4.1	1.3	2.6	4.2	4.2	4.2	5.4	4.5	2.7	2.7	2.7	2.7	2.7	2.7	4.1	4	5.3	5.1	1.4	2.7	2.5	5	4.9	1.2	1.2	2.3	2.3	2.3	2.4	2.6	2.6	2.6	1.3	100	100	99.4	81.6	81.6	81.2	83	76.2	4.9
LEY	1.3	1.4	1.4	2.7	5.3	5.3	4.1	4.1	1.3	2.6	4.2	4.2	4.2	5.4	4.5	2.7	2.7	2.7	2.7	2.7	2.7	4.1	4	5.3	5.1	1.4	2.7	2.5	5	4.9	1.2	1.2	2.3	2.3	2.3	2.4	2.6	2.6	2.6	1.3	100	100	99.4	81.6	81.6	81.2	83	76.2	4.9
C7CDELTA	1.3	1.4	1.4	2.7	5.3	5.3	4.1	4.1	1.3	2.6	4.2	4.3	4.3	5.4	4.5	2.7	2.7	2.7	2.7	2.7	2.7	4.1	4	5.3	5.1	1.4	2.7	2.5	5.1	4.9	1.2	1.2	2.3	2.3	2.3	2.4	2.6	2.6	2.6	1.3	99.4	99.4	100	80.9	80.9	80.5	82.2	76.6	5
HALCYONE	2.6	2.7	2.7	4	3.9	3.9	2.7	2.7	5.1	2.6	2.7	2.8	2.8	2.7	3	2.7	2.7	2.7	2.7	2.7	2.7	4	2.7	3.9	3.8	1.4	2.6	2.5	3.7	2.4	1.2	1.2	3.4	3.4	3.4	3.5	1.3	1.3	1.3	1.2	81.6	81.6	80.9	100	100	92.7	89.5	70.8	3.7
HEATH	2.6	2.7	2.7	4	3.9	3.9	2.7	2.7	5.1	2.6	2.7	2.8	2.8	2.7	3	2.7	2.7	2.7	2.7	2.7	2.7	4	2.7	3.9	3.8	1.4	2.6	2.5	3.7	2.4	1.2	1.2	3.4	3.4	3.4	3.5	1.3	1.3	1.3	1.2	81.6	81.6	80.9	100	100	92.7	89.5	70.8	3.7
SCOTTIE	2.6	2.7	2.7	4	3.9	3.9	2.7	2.7	5	2.6	2.7	2.8	2.8	2.6	2.9	2.7	2.7	2.7	2.7	2.7	2.7	4	2.6	3.9	3.7	1.4	2.6	2.5	3.7	2.4	1.2	1.2	2.3	2.3	2.3	2.3	2.6	1.3	1.3	2.5	81.2	81.2	80.5	92.7	92.7	100	84.2	70.4	3.6
UNITY	1.4	1.4	1.5	2.9	2.8	2.8	1.4	1.4	1.4	1.4	1.5	1.5	1.5	1.4	1.6	1.4	1.4	1.4	1.4	1.4	1.4	2.9	2.8	2.8	1.3	1.5	1.4	1.3	1.3	1.3	1.3	1.3	0	0	0	0	0	1.4	1.4	0	83	83	82.2	89.5	89.5	84.2	100	70.4	3.9
TRIPP	2.6	2.6	2.7	4	3.9	3.9	2.7	2.7	2.5	2.6	4.1	4.2	4.2	3.9	4.4	1.3	1.3	1.3	1.3	1.3	1.3	7	5.3	3.9	5	2.8	5.2	4.9	6.2	4.8	4.6	4.6	2.3	2.3	2.3	2.3	1.3	2.5	2.5	3.7	76.2	76.2	76.6	70.8	70.8	70.4	70.4	100	3.6
API480	4.1	4.3	4.3	4.3	5.6	5.6	5.7	5.7	4	8.2	4.4	4.4	4.4	4.2	4.7	5.7	5.7	5.7	5.7	5.7	5.7	5.7	7	4.2	4	4.5	5.6	5.3	5.3	5.2	11.2	11.2	9.8	9.8	9.8	10	5.5	13.6	13.6	5.3	4.9	4.9	5	3.7	3.7	3.6	3.9	3.6	100

Table S4: Function and distribution of all API480 genes (n=19) shared with *P. larvae* phages, according to Phamerator. For each API480 gene product (pham) that is shared, the number of phages per cluster that have protein homologs are listed. The total number of phages that belong to each cluster are in brackets. Below, the percentage of phages for each cluster that shared proteins with API480 is provided.

API480 GENOME						
Protein	Function	Fern (n=30)	Harrison (n=2)	Vegas (n=7)	Lily (n=1)	Halcyone (n=8)
gp17	Holin	30	2	7	1	0
gp18	Endolysin	30	2	7	1	0
gp19	Hypotethical protein	0	0	4	0	0
gp20	Hypotethical protein	1	2	2	0	0
gp21	Holin	30	2	7	1	0
gp30	Hypothetical protein	0	0	4	0	0
gp34	Hypothetical protein	0	0	0	0	8
gp38	Hypotethical protein	6	0	0	3	3
gp37	Hypotethical protein	0	0	2	0	7
gp39	dUTP diphosphatase	0	0	0	0	7
gp40	Hypotethical protein	0	0	4	0	0
gp41	Hypotethical protein	0	0	4	0	0
gp42	Hypotethical protein	0	0	0	0	1
gp48	Dingle stranded binding protein	0	2	0	0	0
gp50	Host-nuclease inhibitor	0	2	0	0	0
gp52	Hypotethical protein	1	0	2	0	0
gp53	Hypothetical protein	0	2	2	0	1
gp55	Hypothetical protein	11	2	2	1	0
gp73	Hypothetical protein	1	2	6	0	0
		**19 %**	**47 %**	**39 %**	**37 %**	**18 %**

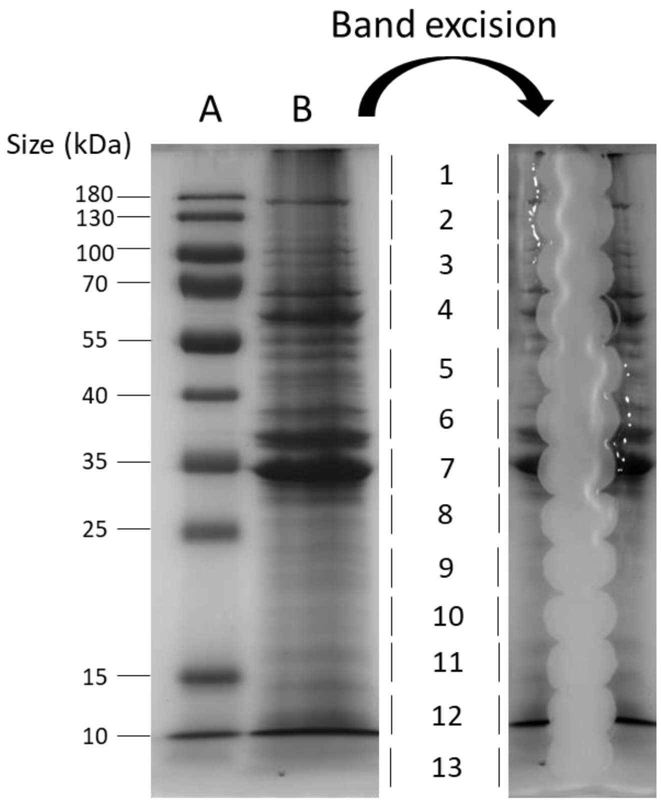

Figure S1: SDS-PAGE analysis of the API480 phage proteins (lane B) on a 12% SDS-PAGE separation gel. Lane A shows the protein ladder bands from10 to180 kDa. The entire lane B was cut into 13 slices, numbered on the right. Band excision is illustrated in the cropped image from the same gel, as indicated.

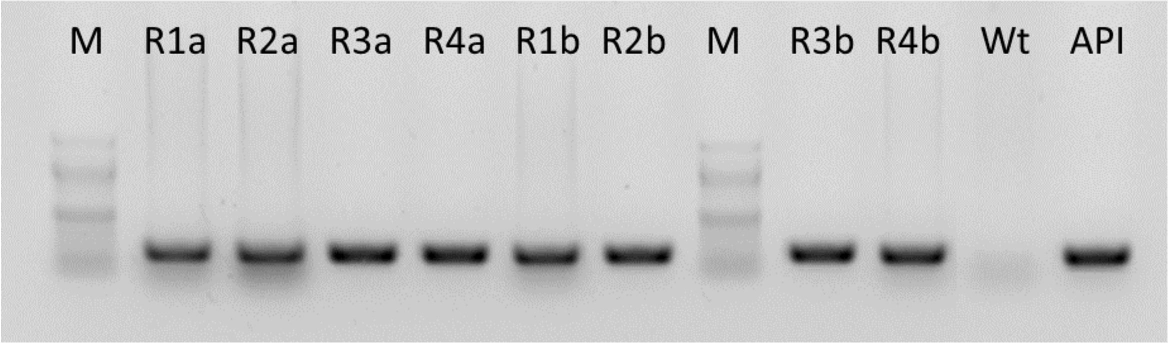

Figure S2: Amplification of CDS_12 fragment. Agarose gel revealing 227 bp bands in R-Pl27 strains tested (R1, R2, R3 and R4 in duplicate (a and b), in API480 (API) and it absence in the original Pl02-27 strain (Wt). DNA ladder 100 bp NEB (M).

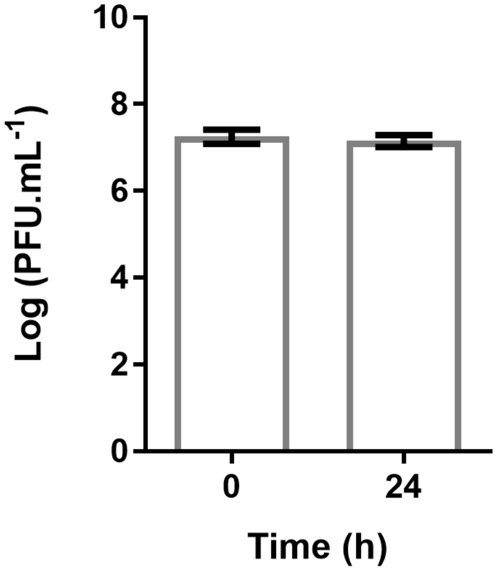

Figure S3: Effect of 50% (w/v) sucrose on API480 phage concentration ($PFU.mL^{-1}$). Data shows represents the average of three independent assays and error bars indicate the standard deviation. LOD (Limit of Detection) = 3 Log. Statistical significance, $p<0.05$.
